# Tool-use practice induces changes in intrinsic functional connectivity of parietal areas

**DOI:** 10.3389/fnhum.2013.00049

**Published:** 2013-02-26

**Authors:** Kwangsun Yoo, William S. Sohn, Yong Jeong

**Affiliations:** Laboratory for Cognitive Neuroscience and NeuroImaging, Department of Bio and Brain Engineering, Korea Advanced Institute of Science and TechnologyDaejeon, South Korea

**Keywords:** tool-use, practice, functional connectivity, resting state, parietal areas, plasticity

## Abstract

Intrinsic functional connectivity from resting state functional magnetic resonance imaging (rsfMRI) has increasingly received attention as a possible predictor of cognitive function and performance. In this study, we investigated the influence of practicing skillful tool manipulation on intrinsic functional connectivity in the resting brain. Acquisition of tool-use skill has two aspects such as formation of motor representation for skillful manipulation and acquisition of the tool concept. To dissociate these two processes, we chose chopsticks-handling with the non-dominant hand. Because participants were already adept at chopsticks-handling with their dominant hand, practice with the non-dominant hand involved only acquiring the skill for tool manipulation with existing knowledge. Eight young participants practiced chopsticks-handling with their non-dominant hand for 8 weeks. They underwent functional magnetic resonance imaging (fMRI) sessions before and after the practice. As a result, functional connectivity among tool-use-related regions of the brain decreased after practice. We found decreased functional connectivity centered on parietal areas, mainly the supramarginal gyrus (SMG) and superior parietal lobule (SPL) and additionally between the primary sensorimotor area and cerebellum. These results suggest that the parietal lobe and cerebellum purely mediate motor learning for skillful tool-use. This decreased functional connectivity may represent increased efficiency of functional network.

## Introduction

Humans develop and manipulate tools in everyday life. Current knowledge on the neural substrates and functions of tool use is mainly derived from patients with apraxia (Goldenberg and Hagmann, 1998). Apraxia is a disorder of motor control characterized by impairment in performing meaningful movement without any impaired basic motor function. Beginning with a study by Hugo Liepmann, numerous studies have shown that there are several types of apraxia such as ideomotor and ideational/conceptual apraxia (Liepmann, 1900, 1908). Studies of apraxia patients have also shown diverse functional impairment in manipulating a tool (Heilman, 2003; Jeong, 2009). Tool-use is not a single cognitive domain and several types of tool-use impairments occur (Goldenberg and Spatt, 2009). In particular, patients with a lesion in the parietal area have difficulties manipulating tools and pantomiming tool-use execution rather than loss of the conceptual aspects (semantic knowledge and basic purpose) of a tool (Vingerhoets, 2008; Goldenberg, 2009), whereas other patients lose the conceptual idea of tools (Johnson-Frey, 2004). Goldenberg and Spatt (2009) showed that impaired retrieval of functional knowledge is related to frontal lesions including inferior frontal gyrus (IFG). In contrast, parietal lesions including supramarginal gyrus (SMG) and superior parietal lobule (SPL), affect actual use of a tool (Goldenberg and Spatt, 2009). Functional imaging studies in a healthy population also support this finding (Martin et al., 1996). Brain regions that support actual skillful tool manipulation (how to use a tool) and regions that store conceptual aspects of a tool (what a tool is for) are different. Previous studies have reported that the cerebellum, IFG, premotor area (PM), SPL, and inferior parietal lobule (IPL), which is subdivided into the SMG and angular gyrus (AG), have specific tool-use functions (Obayashi et al., 2001; Järveläinen et al., 2004; Maravita and Iriki, 2004; Obayashi, 2004; Johnson-Frey et al., 2005; Holmes et al., 2007). Parietal areas and PM support skillful tool-use, whereas the IFG stores semantic knowledge for tool-use (Lewis, 2006). The cerebellum contains an internal model for tool-use (Fogassi and Luppino, 2005; Vingerhoets, 2008; Ramayya et al., 2010).

After the first demonstration of synchronized low-frequency fluctuations (~0.08 Hz) in the resting brain with blood-oxygenation level dependence (BOLD) functional magnetic resonance imaging (fMRI), the “resting state network” and “intrinsic functional connectivity” have received a lot of attention (Biswal et al., 1995; Raichle et al., 2001; Deco et al., 2011; Raichle, 2011). Previous resting state functional magnetic resonance imaging (rsfMRI) studies have shown that anatomically segregated but functionally related brain regions are integrated and are organizing networks (De Luca et al., 2006; Chen et al., 2008). Functional MRI is an indirect measure of neuronal population activity in the brain. This method has given results compatible with EEG or MEG studies. More specifically, it has been shown that resting state network via fMRI is highly related to EEG microstates (Britz et al., 2010). Network organization in the brain has been demonstrated not only with fMRI, but also using structural MRI, DTI, EEG, or MEG (Stam, 2004; Hagmann et al., 2008; Van de Ville et al., 2010). Even though several neuroimaging methods deal different temporal and spatial resolution, and mechanism, multiple studies using different methods have commonly reported that the brain network as a whole, has small-worldness and functionally related regions are highly synchronized (Stam, 2004). In addition, functional connection strength of this synchronization would reflect cognitive or motor performance (Hampson et al., 2006; Baldassarre et al., 2012), maturation (Fair et al., 2008; Dosenbach et al., 2010), and recent experience (Tambini et al., 2010). Resting functional connectivity via fMRI might be more appropriate to study long-term changes because fMRI brain network is relatively more stable compared to the networks from other modalities such as EEG microstates which shows rapid dynamics (Britz et al., 2010).

In addition to the use of resting functional connectivity for representing current brain function, resting functional connectivity has also been shown to have the plasticity. Neuroimaging studies have demonstrated that motor learning induces not only an alteration in brain structure or functional activation with the task but also affects the intrinsic functional connectivity of the resting brain (Büchel et al., 1999; Grigg and Grady, 2010; Voss et al., 2010; Ma et al., 2011; Taubert et al., 2011) as well as structural connectivity (Scholz et al., 2009; Taubert et al., 2010). In addition, with cumulated evidence, it is now broadly believed that functional properties (activation or functional connectivity) and structural properties (cortical thickness or structural connectivity) can be an indirect representation of each other (Ilg et al., 2008; Greicius et al., 2009; Honey et al., 2009; Granert et al., 2011). Hence researchers have been extensively using resting state fMRI to investigate resting functional connectivity representing brain cognitive function and brain structure.

Proficiency at manipulating a new tool is a type of motor learning (Berti and Frassinetti, 2000; Ishibashi et al., 2002a,b; Wolpert et al., 2011). Effect of motor learning on functional connectivity has been extensively reported, however, no study has investigated the effect of tool manipulating practice on functional connectivity in the resting brain. In this study, we chose chopsticks-handling with the non-dominant hand as the tool-use task and demonstrated what and how the functional connectivity changes in the resting brain reflect tool-use performance improvement. Because participants were already adept at chopsticks-handling with their dominant hand, non-dominant hand practice would influence the actual motor skill without affecting the concept of chopsticks. As we mentioned, tool-use can be divided into two sub-domains, conceptual aspect and actual motor skill. In this study, we focused on acquiring motor skill without influencing the conceptual aspect. By doing this, we could extend our knowledge on motor skill aspects of tool-use, and could better understand the changes of the resting brain when acquiring new skill for tool manipulation. Thus, we hypothesized that practicing chopsticks-handling with non-dominant hand would mainly alter the intrinsic functional connectivity of parietal areas.

## Materials and methods

### Participants

Eight healthy young adults [two females; age of 16.5 (*SD*: ± 0.53) years, ranging from 16 to 17 years] volunteered for this study. Every participant was right-handed according to the Edinburgh Handedness Questionnaire Inventory (Oldfield, 1971). We set a score of +40 as a criterion for right-handedness and scores of every subject were over +40. Subjects are ordinary high school students extraneous to any kind of sports, musical skill, or any other hand skills. They also had no history of neurological or psychiatric disorders. The participants and their caregivers gave written informed consent to participate in this study. This study was approved by the Institutional Review Board of Korea Advanced Institute of Science and Technology (KAIST).

### Experimental procedure

**Figure 1:**
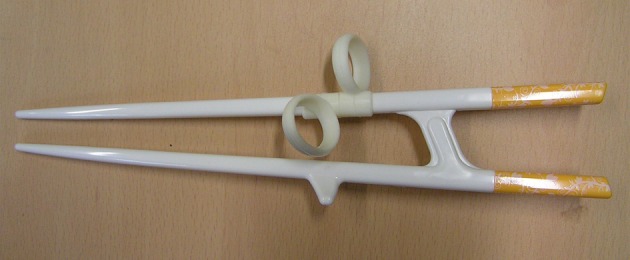
**A chopsticks-aid used for practice and during MRI scanning.** Figure shows a chopsticks-aid for left-handedness, consisting of a plastic body part and two rubber rings to place the index and middle finger. The two sticks are connected, unlike normal chopsticks.

Participants practiced handling chopsticks with their non-dominant hand (left hand) for 8 weeks using a chopsticks aid (Figure 1). Specifically, they practiced moving beans sized <1 cm in diameter from one dish to another using the aid at least 30 min everyday; they also used real chopsticks with their non-dominant hand at every meal (three times per day) during the practice period. Initial performance of chopsticks-handling was measured before starting the practice runs, and the final performance was measured after finishing practice of 8 weeks. Chopstick handling performance was quantified by counting the number of beans moved from one dish to another in 1 minute.

The participants underwent MRI scanning before and after practice, at weeks 0 and 8, respectively. We acquired structural T1 and fMRI data. The fMRI scanning included two sessions, first in a resting state and the latter in performing task. During the resting state session, subjects were asked to stay calm without thinking about anything for 330 s, whereas during the task session, subjects were asked to perform the chopstick-handling action using their left hand for 30 s following a 30 s resting interval, repeated five times for 300 s. Subjects were instructed to keep their eyes open with watching a fixed white cross on the center of black screen during the resting session. For the chopsticks-handling task, subjects were holding a MR-compatible chopsticks aid made of plastic (Figure 1) with their left hand. They performed simple picking and releasing action with this aid, repeatedly.

### MRI acquisition and pre-processing

Structural and functional MRI data were obtained using a 3T MRI scanner (ISOL Technology, Seoul, Korea) at the KAIST fMRI Center. An anatomical T1-weighted MRI was acquired for each subject [repetition time (TR) = 2800 ms, echo time (TE) = 14 ms, flip angle = 60°, field of view (FOV) = 220 × 220 mm^2^, matrix size = 256 × 256, slice thickness = 4 mm, 35 axial slices]. Functional T2^*^-weighted images were also acquired using a gradient echo planar sequence sensitive BOLD signal (TR = 3000 ms, TE = 35 ms, flip angle = 80°, FOV = 220 × 220 mm^2^, matrix size = 64 × 64, slice thickness = 4 mm, 35 axial slices). Functional MRI pre-processing (slice-timing correction, realignment, co-registration, normalization, and smoothing) was performed using Statistical Parametric Mapping (SPM) 8.0 in MATLAB R2011a (7.12) (Natick, MA, USA). The first two fMRI volumes were discarded for signal stabilization before pre-processing. A slice timing correction was performed for the fMRI time series, and spatial realignment was applied to these images. Then, the corrected and realigned fMRI time series images were co-registered with T1 MRI. T1 data were used as a normalization source image to register the fMRI images into the Montreal Neurological Institute space (MNI-152 stereotactic template). Transformation matrices from individual T1 to the MNI-152 T1 stereotactic template were calculated, and these matrices were applied to each co-registered fMRI image. Finally, normalized fMRI images were smoothed using an isotropic Gaussian kernel of 6 mm full-width at half maximum to increase the signal to noise ratio.

### Independent component analysis (ICA) and second level comparison: resting state fMRI

Group ICA was performed on week 0 and 8 rsfMRI data separately using the Group ICA of fMRI Toolbox (GIFT v1.3i). We extracted 20 group-independent components, only 1 of which was selected as a component of interest (sensory motor network), based on its spatial distribution in the brain, covering the primary sensory area (S1), primary motor area (M1), and the supplementary motor area (SMA) (Beckmann et al., 2005; De Luca et al., 2006). Individual resting state sensory motor network (rsSMN) components extracted from group ICA were first converted into z-score maps. Then, we used these pairs of rsSMN *z*-score maps as an input for the two-sample paired *t*-test (week 0 vs. week 8, *p* < 0.001, size > 8 voxels). As a control of task-selectivity in resting state networks, we compared the default mode networks (DMN) of weeks 0 and 8 to determine whether motor practice affected other networks. We selected one component which centers on the precuneus and posterior cingulate cortex, and additionally covers medial prefrontal cortex and lateral parietal areas, as DMN (Raichle et al., 2001). Then, the same procedure was applied to DMN for the second level comparison.

### Task fMRI for selecting regions of interest (ROIs)

For this, we analyzed task fMRI data of week 0 and week 8 altogether collectively. The task fMRI data were analyzed using FMRI Expert Analysis Tool (FEAT) version 5.98, which is a part of the FMRIB Software Library (FSL). We performed GLM analysis for every individual data of week 0 and 8. Then we did group analysis to find common activation areas by chopsticks-handling task both before and after practice; 8 subjects × 2 times, total 16 data set. Z statistic images were thresholded using clusters determined by *Z* values > 4.0 and a (corrected) cluster significance threshold of *P* = 0.05 (Worsley, 2001). By doing this, we could make one group-averaged task activation map for chopsticks-handling. We selected tool-use-related brain regions based on our task activation results and rsSMN, and then drew 5 × 5 × 5 cubic regions of interest (ROIs) in these regions manually. Additionally, we compared the patterns of task activation during chopsticks-handling before and after practice of 8 weeks with the same threshold as above.

### Analysis for the intrinsic functional connectivity within tool-use-related areas

We first extracted the average time series from each ROI determined previously. A pair (weeks 0 and 8) of time-series was obtained for each subject. A functional connectivity analysis was performed for every pair of the time-series using Pearson's correlation within each set individually. As a result, 16 (8 subjects × 2 time points) matrices of correlation coefficients were acquired. Then we transformed the correlation coefficient “*r*” into “*z*” scores by applying Fisher's *r* to *z* transformation. We performed a paired *t*-test group comparison of the time-series correlation using these “z” score matrices. *P*-values < 0.05 (uncorrected) were considered significant.

## Results

### Performance improvement

We performed two-sample paired *t*-test to test subjects' performance improvement. Every participant showed improvements in chopsticks-handling performance with their non-dominant left hand following practice. This improvement was statistically significant on the group level (*p* < 0.0001). Before practice, they moved 4.9 (SD: ± 4.7) beans per minute, whereas they moved 11.8 (SD: ± 5.8) beans per minute after practice, thus showing an increase of 7.1 (SD: ± 2.2) beans per minute).

### Decreased connectivity within rsSMN

**Figure 2:**
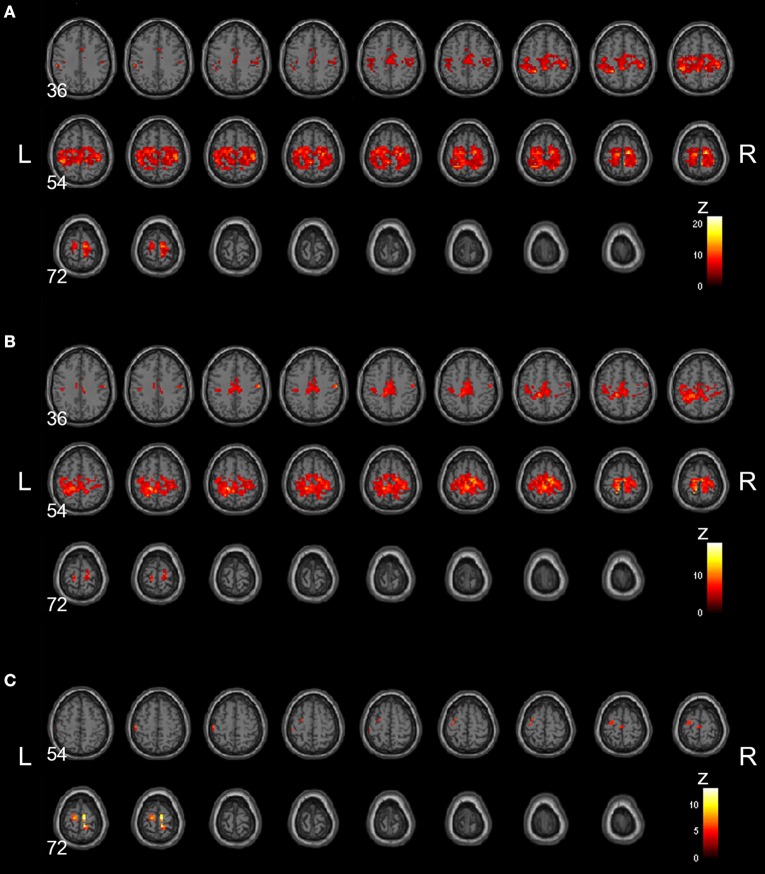
**Group-averaged rsSMNs at week 0 and 8.** Group-averaged rsSMNs were acquired at **(A)** week 0 and **(B)** week 8. Both networks included bilateral primary sensory motor areas and the supplementary motor area. **(C)** Intra-rsSMN connectivity decreased following 8 weeks of practice (*p* < 0.001, size > 8 voxels). The regions affected were the supplementary motor area, the primary sensory area, and the primary motor area in the right hemisphere and the primary motor area and premotor area in the left hemisphere (Neurological view).

**Figure 3:**
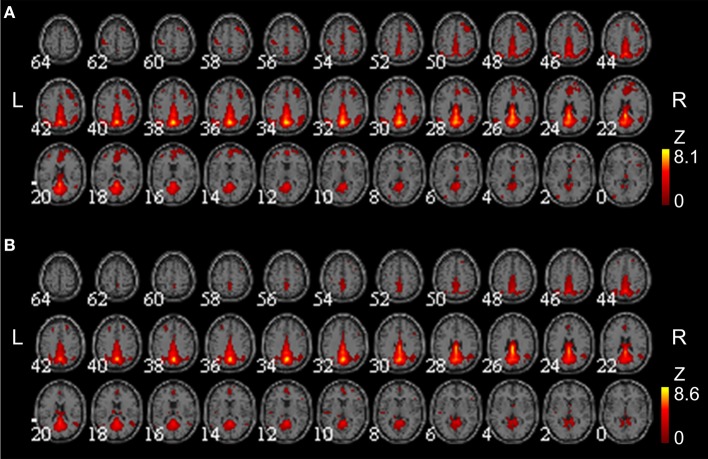
**Group-averaged DMNs at week 0 and 8.** Group-averaged DMNs were acquired at **(A)** week 0 and **(B)** week 8. DMNs centered on the precuneus and posterior cingulate cortex, and additionally covered medial frontal areas and lateral parietal areas.

Group-averaged rsSMNs of weeks 0 and 8 are shown in Figures 2A and B, respectively. In both states, group-averaged rsSMNs commonly included the bilateral S1 and M1. These networks also extended anteriorly to the PM and the SMA and posteriorly to the SPL and SMG. However, the intra-connectivity of the rsSMN at week 8 decreased significantly compared to that at week 0 (*p* < 0.001, size > 8 voxels, Figure 2C). We found decreased M1 and PM connectivity in the left hemisphere and decreased SMA, S1, and M1 connectivity in the right hemisphere at week 8. However, we could not find any significant change in group-averaged DMNs between weeks 0 and 8 (no significance under *p* < 0.001, size > 8 voxels). Figure 3 shows the group-average DMNs of week 0 and week 8.

### Selected motor-related ROIs and task fMRI activation

**Figure 4:**
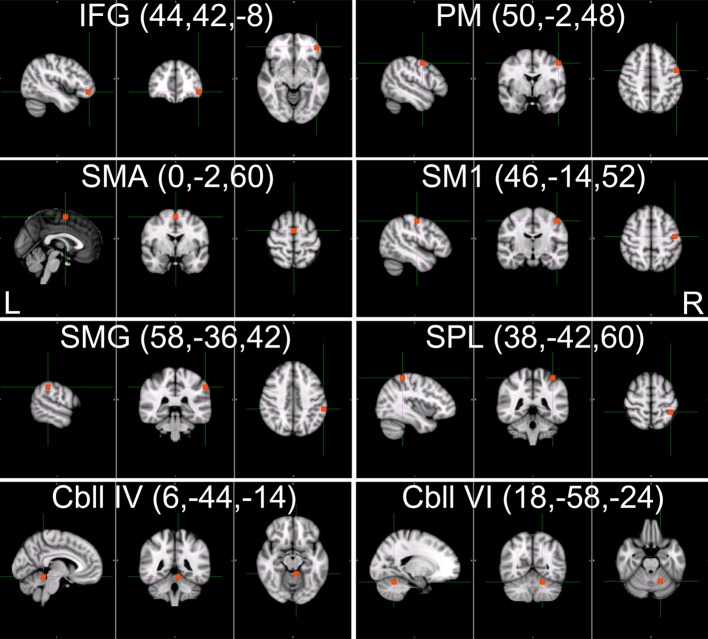
**The 15 selected tool-use-related ROIs.** Fifteen cubic ROIs composed of 5 × 5 × 5 voxels were determined. Bilateral IFG, SMG, SPL, PM, SM1, Cbll VI, and Cbll IV, and SMA were picked for ROIs. Only ROIs in the right hemisphere are shown for the bilateral ROIs. The left hemispheric ROIs are in symmetrical locations. (IFG, inferior frontal gyrus; SMG, supramarginal gyrus; SPL, superior parietal lobule; PM, premotor area; SM1, primary sensorimotor area; Cbll VI, cerebellar lobule VI; Cbll IV, cerebellar lobule IV; SMA, supplementary motor area).

**Figure 5:**
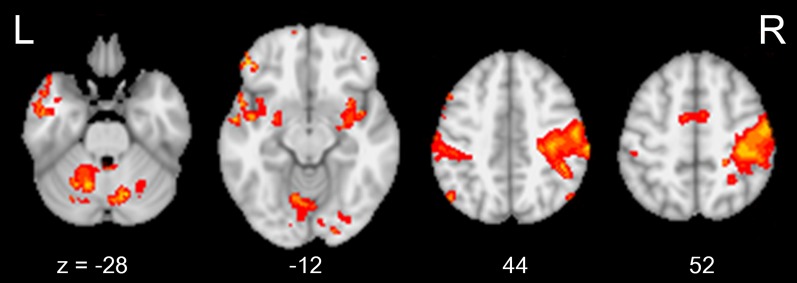
**The brain areas activated during chopsticks-handling.** Figure shows the brain activation induced by chopsticks-handling with a left hand. Activated area includes SM1, PM, SPL, SMG, SMA, IFG, Cbll VI, and Cbll IV. (IFG, inferior frontal gyrus; SMG, supramarginal gyrus; SPL, superior parietal lobule; PM, premotor area; SM1, primary sensorimotor area; Cbll VI, cerebellar lobule VI; Cbll IV, cerebellar lobule IV; SMA, supplementary motor area).

We defined 15 cubic ROIs composed of 5 × 5 × 5 voxels based on the task fMRI and rsfMRI results (Figures 4 and 5). We made one ROI for medial SMA and other 7 ROIs in each hemisphere. These 7 ROIs were in the IFG, PM, primary sensory motor areas (SM1), SMG, SPL, cerebellar lobule IV, and cerebellar lobule VI. These bilateral ROIs were symmetrically placed.

We could not find any significant difference in task-induced activation between week 0 and week 8 (*p* < 0.05, corrected). Figure 5 shows common brain activation during chopsticks-handling with a left hand in week 0 and 8 (*p* < 0.05, corrected).

### Intrinsic functional connectivity changes within tool-use-related areas

**Figure 6:**
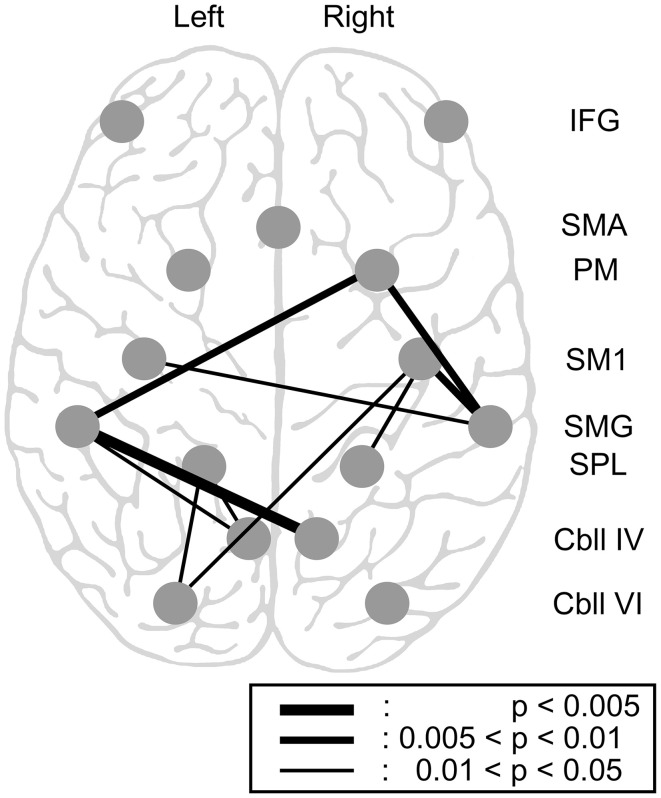
**Changes in intrinsic functional connectivity within the 15 tool-use-related areas.** Pairwise connectivity change following 8 weeks of chopsticks-handling practice is illustrated. Of the 15 regions, parietal areas, particularly the bilateral SMG and SPL, were related to most of the connectivity changes. One connectivity change beyond the parietal lobe was between the left Cbll VI and the right SM1. All significant changes were decreases. (IFG, inferior frontal gyrus; PM, premotor area; SMA, supplementary motor area; SM1, primary sensorimotor area; SMG, supramarginal gyrus; SPL, superior parietal lobule; Cbll IV, cerebellar lobule IV; Cbll VI, cerebellar lobule VI).

We found decreases in intrinsic functional connectivity, whereas no significant increase in functional connectivity was observed after practice (Figure 6). Overall, changes were observed mainly in the right hemisphere. The connectivity between the right SMG and right PM and SM1 decreased, and connectivity between the SPL and SM1 in the right hemisphere also decreased.

The decreased functional connectivity was mainly centered on the parietal area. Specifically, the bilateral SMG had decreased connectivity with the right PM (*p* < 0.01). The left SMG had decreased connectivity with bilateral cerebellar lobule IV (left and right, *p* < 0.05 and *p* < 0.005, respectively), whereas the right SMG had decreased connectivity with bilateral SM1 (left and right, *p* < 0.05 and *p* < 0.01, respectively). The SPL, another parietal area, also showed decreased connectivity with several brain regions; the left SPL had decreased connectivity with the left cerebellar lobules IV and VI (*p* < 0.05), whereas the right SPL had decreased connectivity with the right SM1 (*p* < 0.05). We also found an additional change in connectivity between the right SM1 and the left cerebellar lobule VI, which is beyond the parietal area (*p* < 0.05). However, we did not find any significant changes in functional connectivity in the IFG.

## Discussion

We investigated the effect of practicing skillful tool manipulation, excluding acquisition of the conceptual or semantic knowledge, on intrinsic functional connectivity. We demonstrated that 8 weeks of tool-use practice induced significant decreases in rsSMN connectivity and other intrinsic functional connectivity among brain regions, particularly in parietal areas. Our results support the hypothesis that parietal areas play a role in skillful tool-use manipulation and that this feature is reflected even in a resting state.

Previous studies have showed that intrinsic functional connectivity decreases after long-term practice (Ma et al., 2010, 2011; Voss et al., 2010; Taubert et al., 2011). In these studies, the functional connectivity of the resting brain among task-related regions increases in the early phase of motor learning then decreases in the late phase. These results might suggest and provide evidence for non-linear change of functional connectivity with long-term practice. In addition to functional connectivity, fMRI activation studies also have reported similar patterns, increased in early phase and decreased activation in the late stage of practice (Xiong et al., 2009). This is consistent with the finding of less activation in experts compared to beginners (Bezzola et al., 2012). Previous studies described practice of about 4 weeks as long-term thus 8 weeks practice in our study was assumed as long-term practice. With these previous finding and knowledge, our results provide evidence for that reduced neural cost and enhanced efficiency in the brain's functional network in controlling a cognitive or motor function.

Other studies have reported that resting functional connectivity properties has a significant relationship with task-induced BOLD activity (Mennes et al., 2010, 2011), and that practice induces a decrease in brain activation (Sayala et al., 2006; Reithler et al., 2010). These studies showed that resting state functional connectivity is positively correlated with task-induced brain activation and that long-term practice induces improvement in behavioral performance and decrease in functional activation. These results from literatures coincide indirectly with our results, improvement in chopsticks-handling and decrease in related functional connectivity. In our study, however, we could not see the difference in task-activation pattern between week 0 and 8. This is might be because the task subjects performed in the scanner was too simple. Even though subjects were using chopsticks aid to mimic picking and releasing of chopsticks-handling motion, the task in the scanner was more similar to simple finger-tapping. This task would hardly represent the actual skill of chopsticks-handling. Hence our task-activation result might not reflect chopsticks-handling skill well. Other studies have reported that functional connectivity of the resting-state brain or amplitude of resting functional connectivity may explain variability in subject behaviors (Cole et al., 2012; Zou et al., 2012). It has been also reported that functional connectivity via fMRI can predict maturity of individual brain (Fair et al., 2008; Dosenbach et al., 2010). One study reported a positive association between global efficiency of the functional brain network and intellectual performance (Van den Heuvel et al., 2009). Thus, with the result of improved performance, we suggest that this decreased intrinsic functional connectivity within tool-use-related areas might indicate a change in the network's efficiency for a specific function, becoming more efficient for motor functioning as a subject becomes more proficient at tool-use.

We identified functional connectivity changes in several brain regions, and found that the functional connectivity alterations were centered in the parietal lobe. This result provides evidence for plastic change in parietal areas as the key feature during tool-use practice. The SMG, an anterior part of the IPL, is involved in various aspects of motor function. Actual tool-use execution (Higuchi et al., 2007), imagination, and pantomiming of tool execution (Imazu et al., 2007), and, more specifically, chopsticks-handling (Imazu et al., 2007; Tsuda et al., 2009) actually induce activation of the SMG. As a result of chopsticks-handling practice, intrinsic functional connectivity in the bilateral SMG decreased significantly. The change in the right SMG occurred because participants used their left hand during tool manipulation practice. In contrast, the change in the left SMG supports the motor function dominance of the left hemisphere. Left hemisphere motor function dominance and tool-use is now well-understood. Johnson-Frey et al. (2005) showed a distributed left hemisphere network for tool-use. This network contains the SMG and AG as well as the PM. Other studies have reported that the SMG is involved in motor execution, mental simulation (Grezes and Decety, 2001), and motor attention (SMG in the left hemisphere) (Rushworth et al., 2001). In addition to the SMG, we found a significant decrease in functional connectivity in the SPL with other brain areas. The SPL is also involved in tool-use (Choi et al., 2001; Inoue et al., 2001). The SPL also plays a role in maintaining internal representations (Wolpert et al., 1998), contralateral coding of imagined body parts (Wolbers et al., 2003), hand grasping (Simon et al., 2002), and observation of actions for acquired motor skill (Calvo-Merino et al., 2005).

To determine whether the change in functional connectivity was task-selective, we investigated functional connectivity within the DMN and of the IFG. The IFG is thought to store conceptual aspects of a tool (Johnson-Frey, 2004). However, we found no changes in connectivity involving the IFG. Participants in this study had already learned to use chopsticks; thus, they would not gain any knowledge of the conceptual aspects with practice. Hence, our results are consistent with previous knowledge about the role of the IFG in tool-use and the storage of semantic knowledge, and with our initial hypothesis. In addition, we did not find any changes in DMN connectivity following chopsticks-handling practice. The DMN is shown to be deactivated during a task and not to be correlated with motor function (Raichle et al., 2001; Damoiseaux et al., 2006). Our result of maintained DMN with practice accords with these previous reports. This supports that resting state networks play a specific role in each cognitive function or that resting state networks have task-selectivity (Harrison et al., 2008).

Our study has several limitations. First, the number of subjects participating in this study was relatively small. Thus, we did not find a correlation between functional connectivity and improved performance. It might be possible to identify this correlation with additional subjects. Second, the participants were relatively young. Thus, their brains may have been more flexible than those of older adults. Several neuroimaging studies have shown age-dependent differences in brain connectivity and plasticity (Damoiseaux et al., 2008; Fair et al., 2008; Power et al., 2010). However, no research has directly compared practice-induced brain plasticity in the resting state connectivity in different age groups. Third, we applied rather less stringent threshold for the analysis of rsSMN than task fMRI analysis to show clear result. This might increase the risk of type-I error. However, in contrast with change in rsSMN, we could not find any change in DMN with the same threshold. Thus, given these results, we carefully suggested that there is a task-selectivity in resting state networks.

In summary, we demonstrated a tool-use practice-induced decrease in functional connectivity in the resting brain. These changes showed network selectivity such that the sensory motor network showed decreased connectivity, whereas connectivity of the DMN remained constant. Our results indicate that parietal regions play a role in skillful manipulation of a tool in the tool-related fronto-parietal network, and that decreased intrinsic functional connectivity represents enhanced neural efficiency for performing a task.

### Conflict of interest statement

The authors declare that the research was conducted in the absence of any commercial or financial relationships that could be construed as a potential conflict of interest.
